# The Influence of DMDBS on Crystallization Behavior and Crystalline Morphology of Weakly-Phase-Separated Olefin Block Copolymer

**DOI:** 10.3390/polym11030552

**Published:** 2019-03-22

**Authors:** Yongsheng Zhao, Cheng Yao, Tao Chang, Yanling Zhu

**Affiliations:** 1 Department of Applied Chemistry, School of Science, Analytical & Testing Center, Northwestern Polytechnical University, Xi’an 710072, China; 2 Shaanxi Provincial Key Laboratory of Papermaking Technology and Specialty Paper, Xi’an 710021, China; chengyao@126.com (C.Y.); taochang.sust@163.com (T.C.); 3 State Key Laboratory of Pulp and Paper Engineering, South China University of Technology, Guangzhou 510640, China

**Keywords:** polymer crystallization, olefin block copolymer (OBC), nucleating agent, DMDBS

## Abstract

Olefin block copolymer (OBC), with its low hard segments, can form unique space-filling spherulites other than confined-crystallization morphologies, mainly due to its weak phase-separation. In this work, 1,3;2,4-Bis(3,4-dimethylbenzylidene) sorbitol (DMDBS), a well-known nucleating agent, was used to tailor the crystallization behavior and crystalline morphology of OBC. It was found that DMDBS can precipitate within an OBC matrix and self-assemble into crystalline fibrils when cooling from the melt. A non-isothermal crystallization process exhibited an increased crystallization rate and strong composition dependence. During the isothermal crystallization process, DMDBS showed a more obvious nucleating efficiency at a higher crystallization temperature. OBC showed typical spherulites when DMDBS was added. Moreover, a low addition of DMDBS significantly decreased the crystal size, while a large addition of DMDBS induced aggregates, due to the limited miscibility of DMDBS with OBC. The efficient nucleating effect of DMDBS on OBC led to an increased optical transparency for OBC/DMDBS composites.

## 1. Introduction

**Scheme 1:**
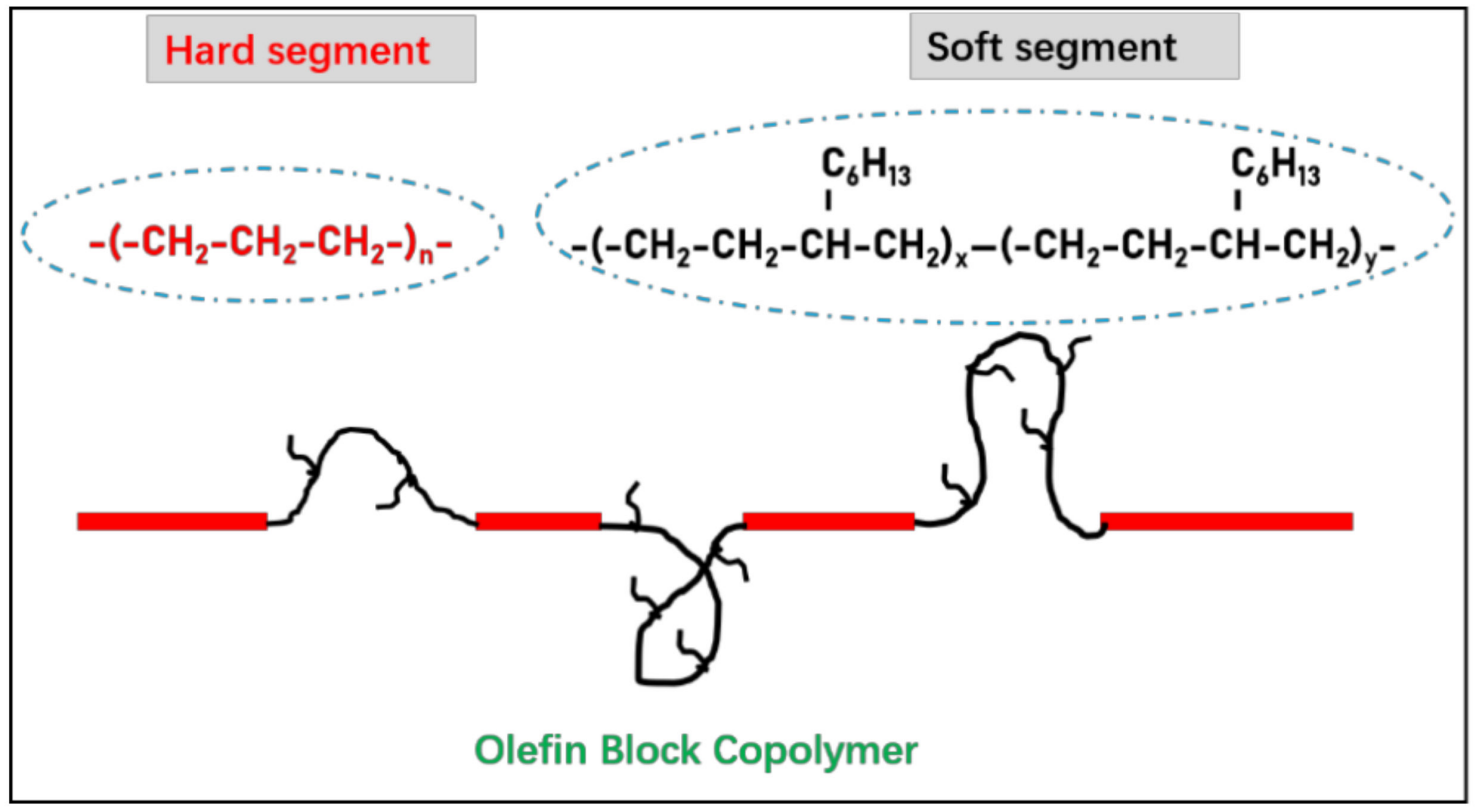
Chain architecture and chemical structure of olefin block copolymer (OBC).

Due to so-called chain shuttling technology, olefin block copolymer (OBC) has been recently synthesized by Dow Chemical Company, and commercialized as a thermoplastic elastomer [1,2,3,4]. As shown in Scheme 1, during the chain growth process, a chain shuttling agent was used to either activate or inactivate the branch incorporation of 1-octene into the chains, resulting in a multiblock copolymer of semicrystalline polyethylene (PE) hard segments, alternating with amorphous soft segments with short branches [5,6]. In addition, the detailed block length and block number could be easily regulated by simply changing the content of the chain shuttling agent [7]. In this way, the varied apparent mechanical properties were thus achieved. Furthermore, OBC possesses better performance and is widely used in many applications, mainly due to the high melting temperature of hard blocks and low glass transition temperature of soft blocks [8].

The structure-property relationship of OBCs has been reported by many researchers. For an OBC system, the dissimilarity between hard blocks and soft blocks is estimated by the 1-octene co-monomer content difference (∆C8), which determines the phase segregation strength [9,10,11]. It has been reported that large ∆C8 could lead to mesophase separation, meaning domain sizes in excess of 100 nm, and confined crystallization behavior [12]. However, most OBCs are in a weakly-phase-separated system, meaning the physical properties of OBCs are dominated by their crystalline morphologies [13,14]. The partial-branched hard segments limit the attainable mechanical properties of pure OBCs. In order to improve the mechanical properties of OBCs, with the aim of broadening its applications, two main approaches were used, including blending with homo-polymers and reinforcing by adding fillers. On the one hand, polypropylene (PP), polyethylene with different molecular weight (PE), poly(lactic acid) (PLA), Kevlar, and PP fibers were blended with OBC to enhance its tensile strength [15,16,17,18,19]. On the other hand, various fillers, such as carbon nanotubes (CNTs), graphene, quaternary ammonium salt functionalized grapheme oxide (CTAB-GO), and nanoclay were added into the OBC to nucleate and promote its crystallization kinetics [20,21,22]. Wu and co-workers investigated the effect of CNTs and graphene on the crystallization kinetics of OBC and found that both CNTs and graphene shorten the induction period of crystallization and increase the crystallization rate of OBC [23]. A modified CTAB-GO led to a better dispersion and showed a stronger nucleation ability than GO and largely improved the mechanical properties of OBC [20]. Fan and co-workers reported that the collapsed organically modified montmorillonite (c-OMMT) exhibited a stronger nucleation ability on crystallization of OBC than intercalated OMMT [24]. Also, the addition of c-OMMT into OBC decreased the crystal size and increased lamellar thickness and the number of tie-molecules between lamellae, resulting in an obvious strain-hardening behavior [25]. Therefore, it is of great importance and highly efficient for OBC to tune its crystalline morphologies in order to tailor its physical properties.

1,3;2,4-bis(3,4-dimethylbenzylidene) sorbitol (DMDBS) is a chiral molecule and can self-assemble into nanofibrils, due to intramolecular hydrogen bonding interactions [26,27]. DMDBS and its derivatives can dissolve in the polymer and form nucleating particles when re-crystallized from the melt [28]. Therefore, DMDBS is widely used as nucleating agent for polypropylene because of their matchable crystalline structures and lattice parameters [29,30,31]. In general, the dissolution and re-crystallization process provides a good dispersion and large specific surface. When DMDBS is added into isotactic polypropylene (iPP) system, a good clarification effect can also be found, especially when the concentration of DMDBS is lower than 1 wt % [31]. Many works have proven that DMDBS significantly increases the number of crystal nuclei and results in a great reduction of the final crystal size, which is strongly related to its highly improved transparency. Also, Lai et.al added DMDBS into poly(vinylidene fluoride) and found that the self-assembly process of DMDBS can be well-tuned by the so-called phase inversion method [32]. However, so far, an OBC/DMDBS composite has not been reported on by other groups.

In this work, we propose a simple and convenient method, by blending the nucleating agent DMDBS with olefin block copolymers, to regulate the crystalline structure and morphology of OBC, which can generally form a dispersed polyethylene crystal or even an interconnected crystalline network embedded in the amorphous rubber network. This work may provide some guidelines on tuning crystalline morphologies of crystalline block copolymers and enrich the knowledge of nucleating effects for thermoplastic elastomers.

## 2. Experimental

### 2.1. Materials

The OBC used in this work is a commercial product provided by Dow Chemical Company. In order to easily detect its crystallization behaviors, OBC with a grade name of Infuse 9530 was selected, since it has a high crystallinity. In addition, it possesses a molecular weight of 78,640, with a polydispersity of 1.97. Its weight fraction for the hard block is 35 wt %, the total 1-octene content is 9.6 mol %. DMDBS was purchased from Milliken Chemicals in powder form, with a code of Millad-3988. In this work, the OBC material we selected had an *χN* value of ~2.6, which waslower than the reported critical value of *χN* for the microphase separation transition of OBCs (*χN* = 4) [12]. Hence, OBC in this work was a weakly-phase-separated system.

### 2.2. Sample Preparation

The OBC pellets and DMDBS powders were melt-blended by an internal mixer at a fixed rotational speed of 60 rpm and a mixing temperature of 190 °C for 10 min. The blended samples containing different contents of DMDBS were prepared and named as OBC-D0.25, OBC-D0.5, OBC-D1, OBCD2.5, and OBC-D5. For example, OBC-D2.5 means 2.5 wt % DMDBS was melted and mixed with OBC. For comparison, the neat OBC was prepared with a same procedure as a reference sample.

### 2.3. Characterization

In order to determine the crystallization and melting behaviors of OBC/DMDBS samples, a Perkin-Elmer diamond-II differential scanning calorimetry (DSC) was used. The samples were first heated to 190 °C to erase the thermal history, then cooled to room temperature at a cooling rate of 10 °C/min. Afterward, the second heating scans up to 190 °C was carried out. The tests were conducted at a constant rate of 10 °C/min under nitrogen atmosphere. The relative crystallinity could be calculated by quantitatively evaluating the melting peak areas of the composites in relation to the standard enthalpy of polyethylene crystals, based on the following equation:

(Xc = Δ*H*_m_/Δ*H*_m_°) (1)

The theoretical heat of melting of polyethylene with 100% crystallinity was Δ*H*_m_° = 292 J/g [16].

Wide-angle X-ray diffraction (WAXD) measurements were performed on a Bruker DISCOVER D8 diffractometer (Bruker, Billerica, Massachusetts, USA) with CuKα radiation. The scanned 2θ range was from 2.5° to 30° with a scanning rate of 0.02°. The samples used for WAXD analysis were thin layers of 1 mm thickness obtained by compression molding. During compression molding, the samples were first heating at 190 °C for 10 min and subsequently underwent fast cooling. After that, the WAXD tests were conducted on these samples at room temperature.

Rheological measurements (MCR301, Anton Paar, Graz, Austria) were conducted in a linear viscoelastic regime using a rotational rheometer with a 25 mm diameter parallel plate geometry. During the tests, samples were heated to 250 °C to ensure complete melting and were subsequently cooled at 5 °C/min while performing small amplitude oscillatory shear test. The linear viscoelastic region was determined by the strain sweeps. The strain amplitude was set as 5%. All the experiments were conducted under nitrogen to minimize the possibility of sample degradation and all temperature ramps were repeated twice to check reproducibility.

Polarizing optical microscopy (POM) was performed using an Olympus BX-51 optical microscope (Olympus, Tokyo, Japan) under crossed polarization and the optical microscope was equipped with DP-27 CCD and a Linkam hot-stage. Small thin layered samples were placed between two microscope cover glasses and placed on the hot stage. The samples were melted at 240 °C for 5 min and then cooled. The morphological photographs were recorded with the aid of a digital camera.

UV-vis spectroscopy (Agilent Cary5000, Agilent, Palo Alto, California, USA) was carried out to collect UV-vis spectra to evaluate the optical properties of the OBC/DMDBS samples. The samples for optical transparency were prepared by mini-injection molding, where the samples were melted at 190 °C and then injected into the cold mold.

Standard dumbbell-shaped samples were prepared by mini-injection molding. The sample size was 25 × 4 × 2 mm^3^. Cyclic tensile tests were performed on a SANS Universal tensile testing machine according to the GB/T528-2009 standard. The strain was set as 300% and ten loading-unloading cycles were conducted. All the tests were conducted at room temperature at a fixed crosshead speed of 50 mm/min and five specimens were tested for each group.

## 3. Results and Discussion

### 3.1. Phase Separation of DMDBS from OBC Melt

**Figure 1:**
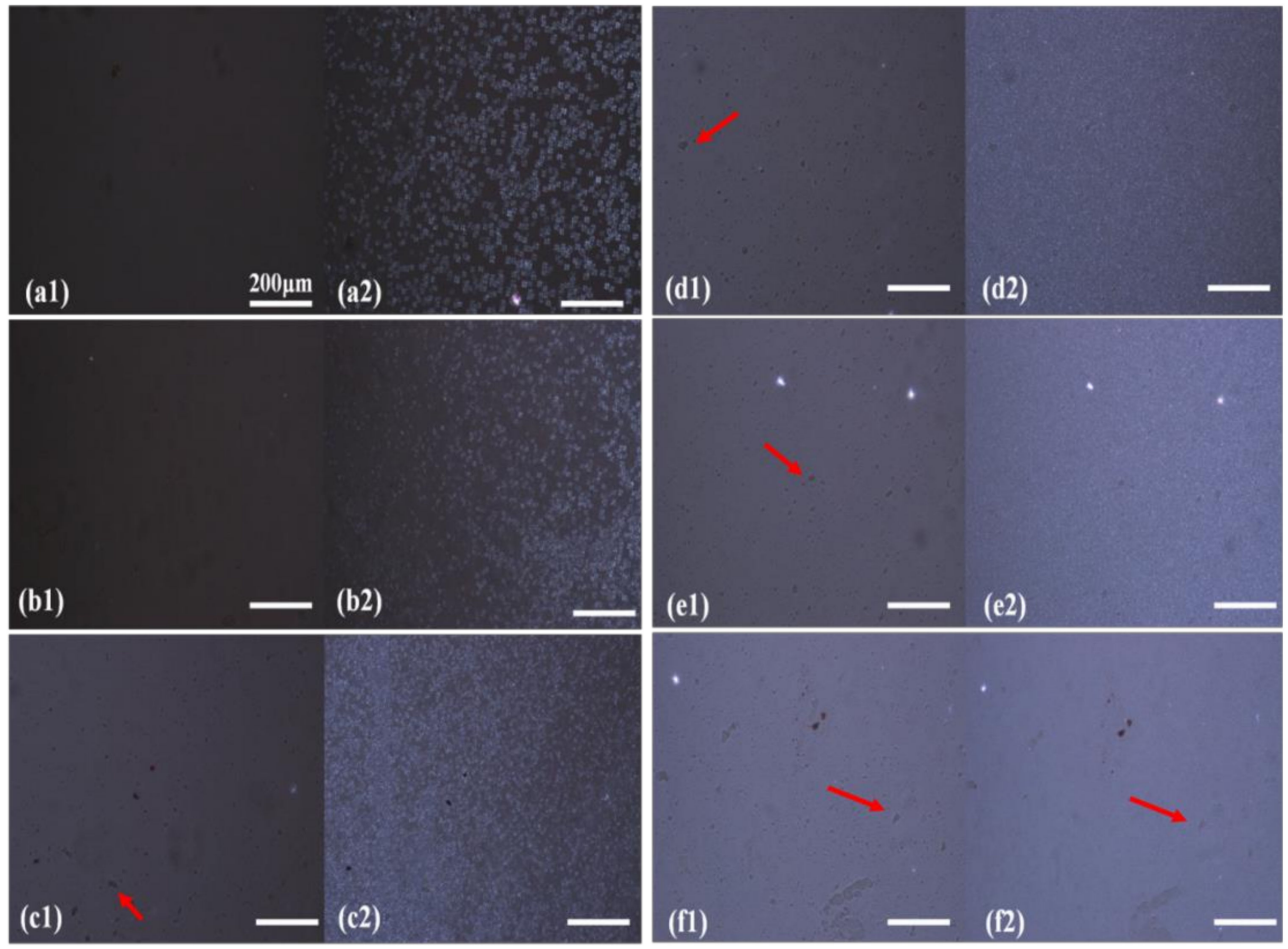
Polarizing optical microscopy (POM) images of olefin block copolymer/1,3;2,4-Bis(3,4-dimethylbenzylidene) sorbitol (OBC/DMDBS) samples at different temperatures: (**a1**). OBC at 190 °C; (**a2**). OBC isothermally crystallized at 116 °C for 10 min; (**b1**–**f1**). OBC/DMDBS samples with different content of DMDBS at 190 °C; (**b2**–**f2**). OBC/DMDBS samples with different content of DMDBS isothermally crystallized at 116 °C for 10 min.

The representative POM images of pure OBC and OBC/DMDBS composites under different temperatures were captured. As shown in Figure 1, when the sample was heated to 190 °C, the neat OBC was totally melted. Figure 1a1 shows that there was no background information, since no crystallization occurred. After the temperature dropped to 116 °C, the hard segments of OBC started to crystallize [2]. Figure 1a2 shows typical spherulites of OBC after having been isothermally crystallized at 116 °C for 10 min. When DMDBS was introduced into OBC system, Figure 1b1 shows that there was still no background information, indicating that a lower addition (0.5 wt %) of DMDBS could mix well with OBC. However, OBC samples containing a higher addition (>0.5 wt %) of DMDBS presented black dots with different sizes, which should be DMDBS aggregates due to the limited miscibility between OBC and DMDBS. After isothermal crystallization, OBC/DMDBS composites showed decreased crystal size with the increase of DMDBS content. 

**Figure 2:**
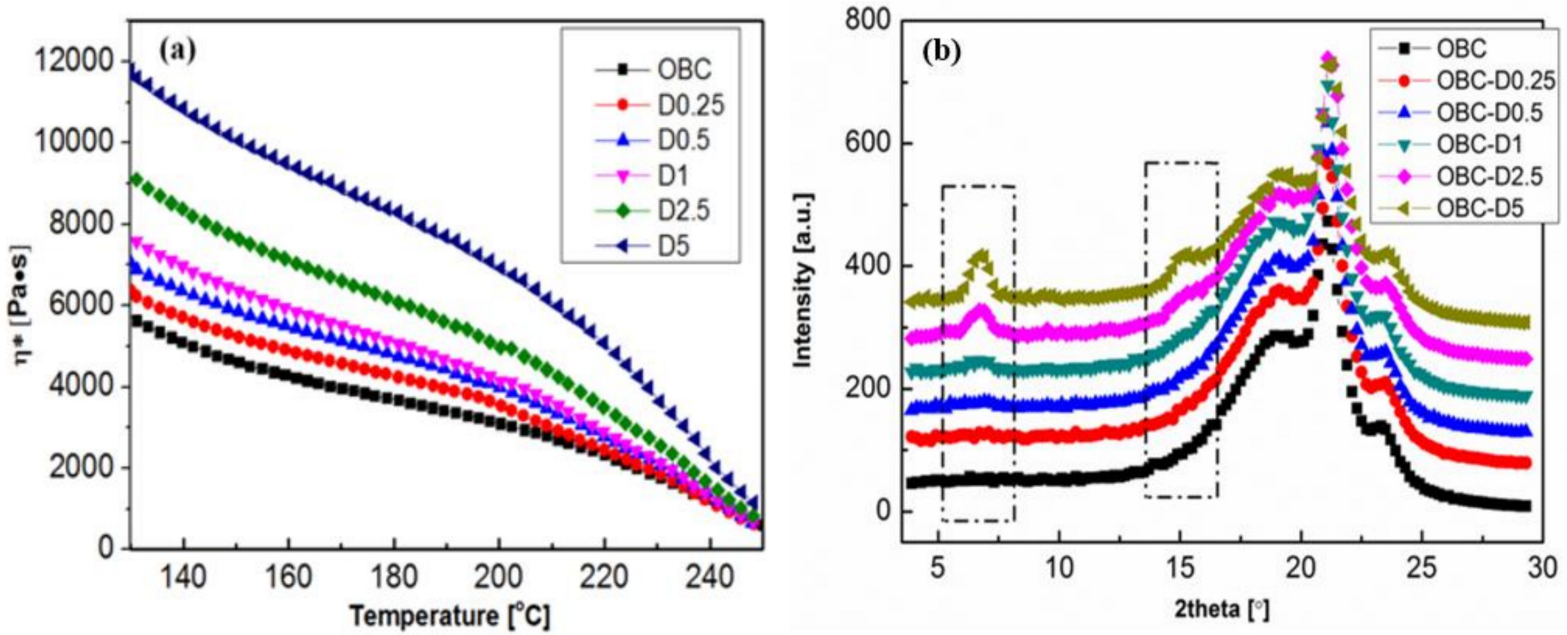
(**a**) Temperature dependence of complex viscosity as a function of DMDBS concentration during cooling at 2 °C/min and after annealing at 260 °C for 10 min; (**b**) 1d-wide-angle X-ray diffraction (WAXD) diffraction intensity as a function of 2θ for OBC/DMDBS composites.

The phase separation behavior of DMDBS and OBC melts can be detected by the oscillatory shear rheological data. Screenivas et al. studied the phase separation temperature of DMDBS from isotactic polypropylene (iPP) through rheological investigations, and reported that the abrupt increase of complex viscosity (η*) was attributed to the result of crystallization of DMDBS into nanofibrils [33,34]. The same experiment was conducted on OBC/DMDBS systems in this work. As can be seen in Figure 2a, there was an obvious increase in complex viscosity (η*) when cooling OBC from 250 °C to 130 °C. Before testing, the samples were heated to 250 °C and held for 5 min to eliminate their thermal history. The temperature dependence of η* was approximately similar for all OBC samples containing different content of DMDBS. With the increase of DMDBS, the complex viscosity of the composites gradually increased. With the decrease of the temperature, the complex viscosity achieved a more abrupt increase during cooling, especially for OBC samples with high contents of DMDBS. As for the pure sample, a slight change of η* was possibly due to the weak mesophase separation of OBC, which has been reported in previous literature [35]. The critical transition of OBC/DMDBS samples was related to the precipitation of DMDBS from the OBC melt. As shown in Figure 2b, the diffraction intensity of OBC/DMDBS as a function of 2θ was presented. As for neat OBC, we can see two obvious peaks located at 21.7° and 24.2°, which can be respectively attributed to the (110) and (200) crystalline planes of polyethylene orthogonal crystals, according to the literature [34]. The introduction of DMDBS had no influence on the crystal form of OBC. With the increase of DMDBS content, diffraction peaks at 6.8° and 14.9° gradually increased, indicating the formation of DMDBS crystals. Overall, combined with POM images of OBC/DMDBS samples at different temperatures, DMDBS precipitated within the OBC matrix and self-assembled into crystalline fibrils when cooling from the melt.

### 3.2. Isothermal and Non-Isothermal Crystallization Behavior of OBC/DMDBS Samples

**Figure 3:**
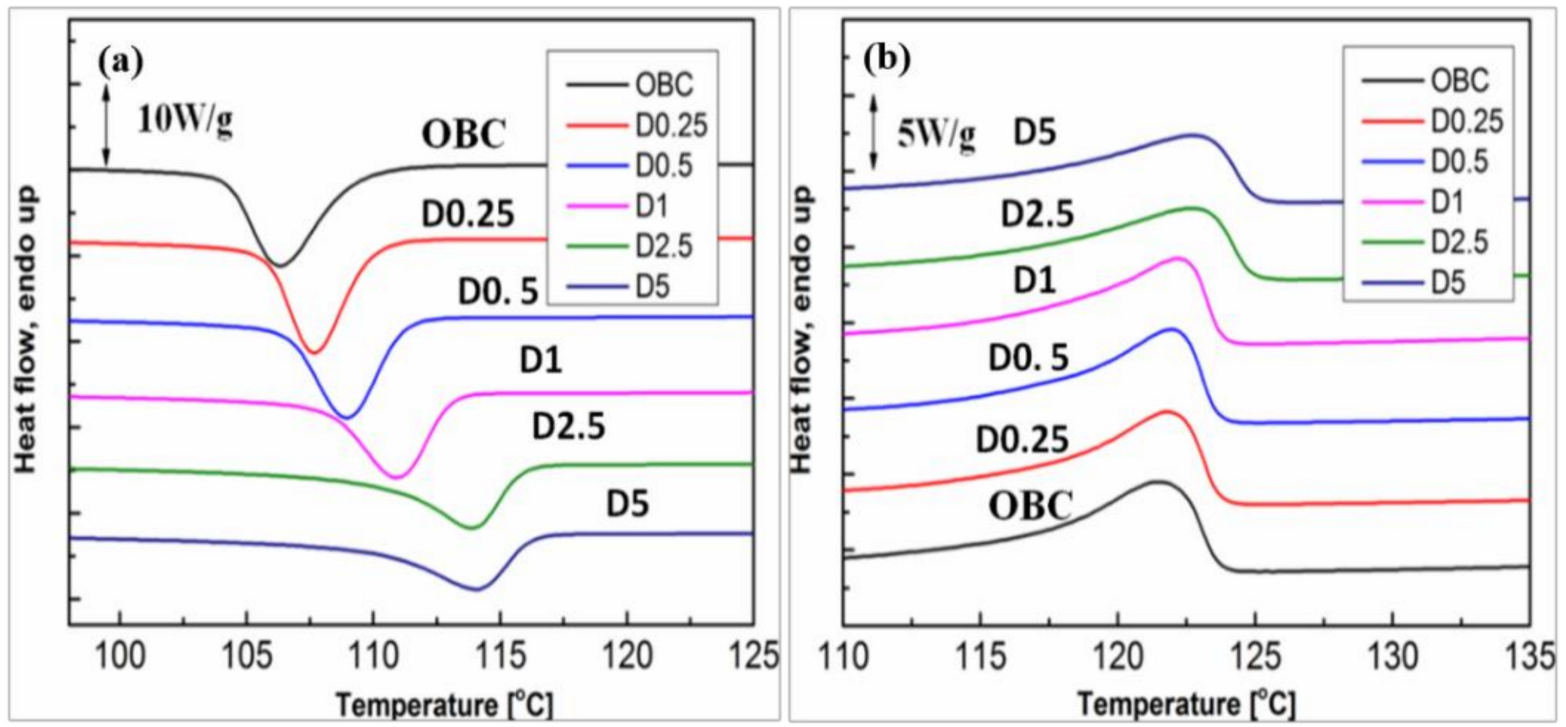
Differential scanning calorimetry (DSC) curves of OBC with and without different dosages of DMDBS (**a**) during the cooling process and (**b**) during the subsequent heating process. Note: Controlled heating and cooling rates of 10 °C/min were applied.

First, the crystallization behaviors of OBC containing different contents of DMDBS were estimated by DSC. Figure 3 presents the non-isothermal cooling and subsequent melting curves of OBC/DMDBS samples containing different contents of DMDBS. It should be noted that DMDBS can nucleate OBC and increase the crystallization temperature. From the perspective of molecular structure, a small amount of 1-octene incorporation into the hard segments for pure OBC led to slowed crystallization and decreased overall crystallinity in comparison to polyethylene [34]. The initial crystallization temperature of OBC is about 112.5 °C and the peak temperature is 106.3 °C. When adding DMDBS, a single exothermic peak is observed and the crystallization is strongly accelerated. Figure 2b shows the melting curves of these OBC/DMDBS samples. Also, a single peak was observed. With the increase of DMDBS content, the melting peak temperature (T_m_) has a slight increase. The introduction of DMDBS also resulted in a broadened melting peak, meaning an increase and wide distribution in the lamellar thickness of PE crystals. 

Table 1 summarizes the melting temperature (T_m_), crystallization peak tdemperature (T_c,p_), enthalpy (ΔH_m_), and the relative crystallinity (X_c_) of OBC/DMDBS samples containing different contents of DMDBS. With the increase of DMDBS content, the crystallization peak temperature increased from 106.3 °C to 114.0 °C, as can be found in Table 1. At the same time, the crystallization peak temperature (T_c,p_) gradually increased with the increase of DMDBS content at low additions, while T_c,p_ was almost unchanged within high DMDBS contents, ranging from 2.5 wt % to 5 wt %, indicating a nucleation saturation effect. In addition, the relative crystallinity was also calculated based on thermal enthalpy during melting. It can be seen that the relative crystallinity (Xc) was nearly the same for the pure OBC and OBC/DMDBS composites.

**Figure 4:**
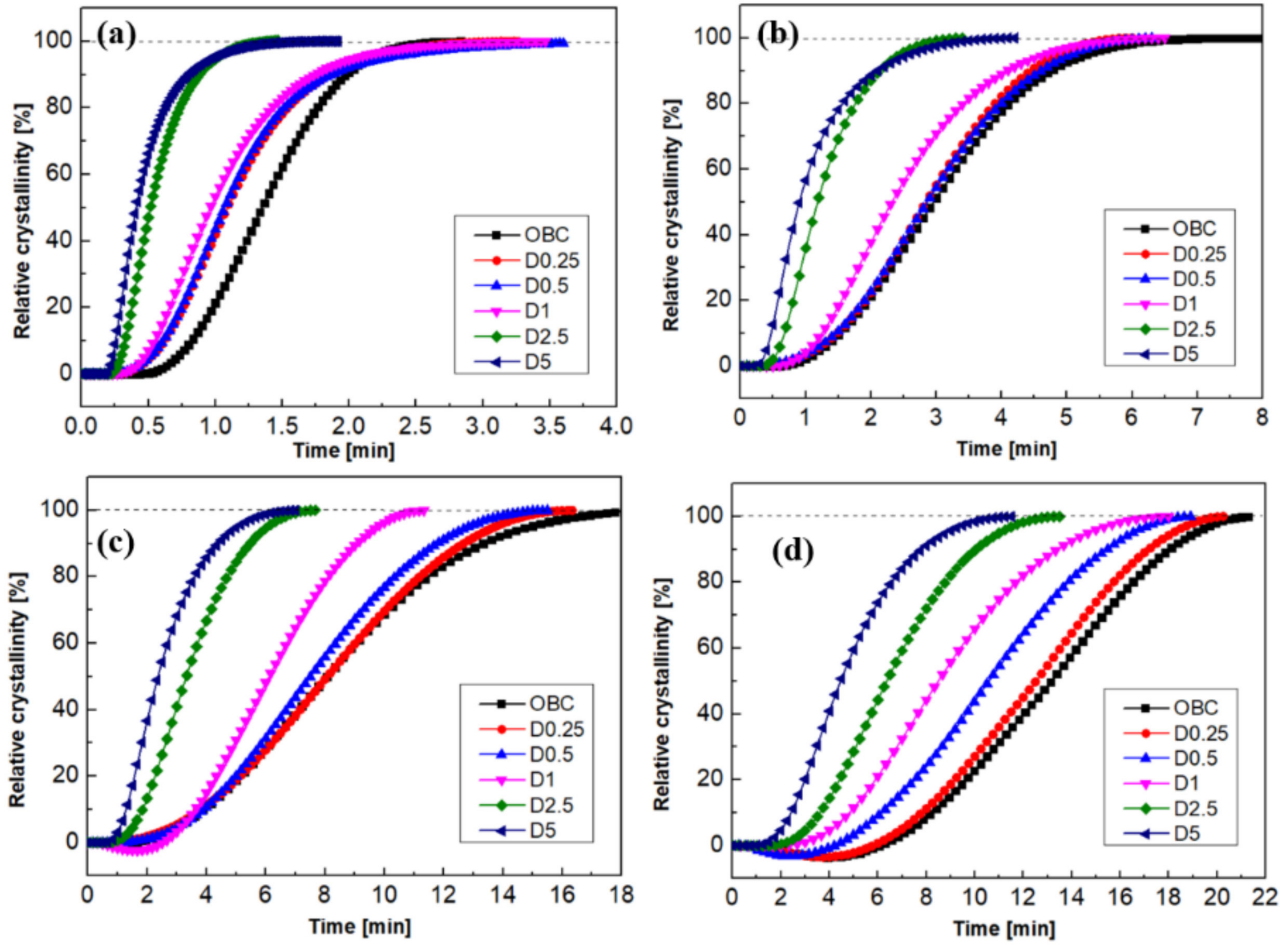
Evolution of the relative crystallinity X_t_ for OBC/DMDBS composites with different DMDBS contents during isothermal crystallization processes at different temperatures of (**a**) 110 °C (**b**) 112 °C (**c**) 114 °C and (**d**) 115 °C.

Figure 4 shows the curves of relative crystallinity (X_t_) of the OBC/DMDBS composites as a function of different contents of DMDBS under different isothermal conditions. It is obvious that adding DMDBS into an OBC matrix increases the crystallization rate. With the increase of DMDBS content, the acceleration effect is more remarkable. The general trend is that, the crystallization of OBC is faster at lower crystallization temperature and only a large addition of DMDBS shows a significant accelerating effect. At higher crystallization temperatures, the crystallization rate of OBC slows down. When adding a small amount of DMDBS, it is obvious to get a fast crystallization process for the OBC system. For the isothermal crystallization processes, the first half of exothermic peak is commonly ascribed to the nucleation through the epitaxy of polymer chains onto DMDBS fibrils, while the other half of peak reflects the crystallization growth via self-diffusion of macromolecules into crystal lattice [36]. It could be seen that the slope increased obviously at the initial stage of crystallization and the crystallization completion was also accelerated, which further indicated that DMDBS mainly acted as nucleation points after being precipitated from the OBC melt.

**Figure 5:**
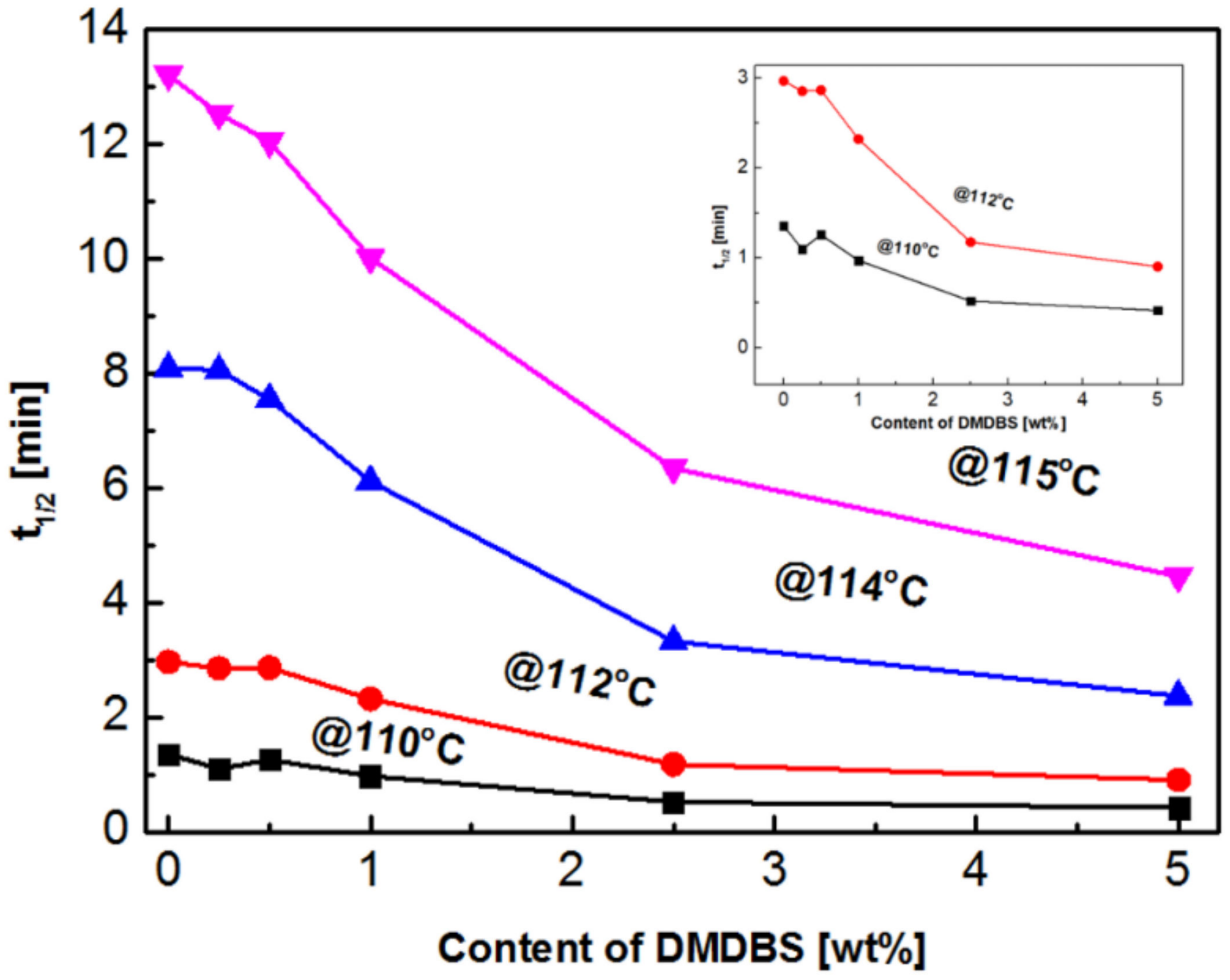
Changes of the crystallization half-time, t_1/2_, as a function of the DMDBS content for OBC/DMDBS composites at different isothermal crystallization temperatures. Note: Inset is a separate image of t_1/2_ as a function of DMDBS content.

Figure 5 shows the isothermal crystallization half-time of OBC/DMDBS composites with different DMDBS contents at different crystallization temperatures. The smaller the crystallization half-time, the faster the crystallization of the samples [37]. The crystallization half-time (t_1/2_) of OBC/DMDBS composite showed a strong dependence on DMDBS content, especially when the crystallization temperature was high. The crystallization process of pure OBC could be completed in a short time when crystallized at 110 °C. After adding DMDBS, the crystallization half-time was further reduced. With the increase of crystallization temperature, the crystallization half-time of OBC increased and the corresponding crystallization rate decreased. In comparison, the crystallization half-time decreased significantly after adding DMDBS. Generally, in the systems with lower DMDBS content (D0.25, D0.5, D1), it was easier to get an acceleration effect from DMDBS at higher crystallization temperatures. However, in the systems with higher DMDBS content (D2.5, D5), the crystallization rate of the OBC composites increased significantly, regardless of the crystallization temperature. 

### 3.3. The Crystalline Morphology of OBC/DMDBS Samples

**Figure 6:**
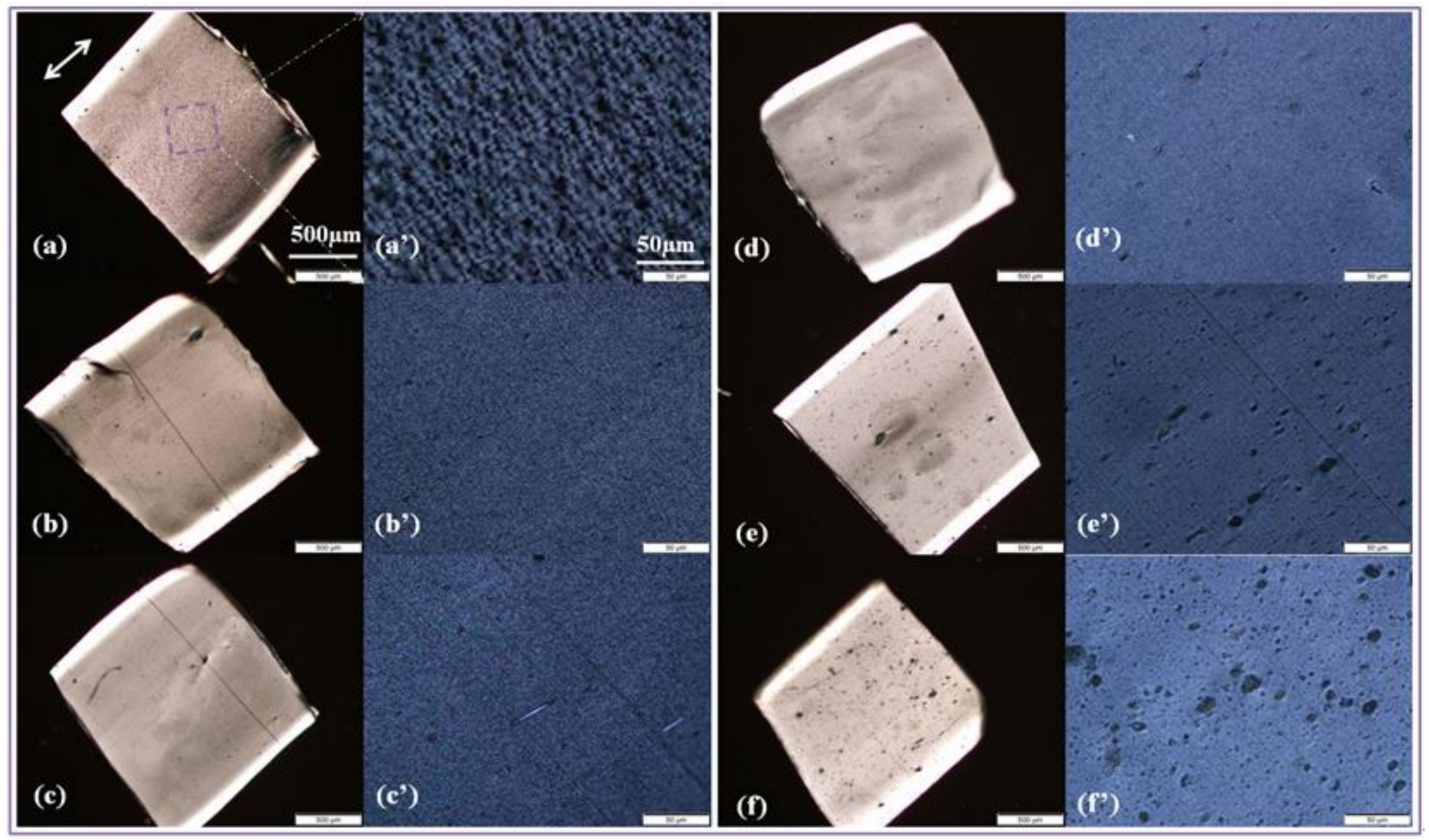
Representative POM images for OBC/DMDBS mini-injection molded samples with different contents of DMDBS. (**a**) OBC; (**b**) D0.25; (**c**) D0.5; (**d**) D1; (**e**) D2.5; (**f**) D5.

In order to get the morphological details, POM images of OBC/DMDBS composites were captured for mini-injection molded samples. Figure 6 presents representative optical images for pure OBC and OBC/DMDBS samples containing different contents of DMDBS. For neat OBC, there was an obvious skin-core structure, mainly due to the temperature gradient [38]. The enlarged image of the core structure shows that the crystals were typically spherulites. The spherulite size was about 10 μm. When 0.25 wt % DMDBS was added, the crystal size of OBC samples decreased significantly and the crystal size was uniform. With the increase of DMDBS content, the crystal size of the sample further decreased and the crystal distribution became denser. However, when the content of DMDBS reached 2.5 wt %, black spherical areas (10–30 μm in size) appeared in the samples. When the content of DMDBS was higher, the black spherical areas increased. These black spherical areas represent aggregates of DMDBS, due to the limited miscibility between OBC and DMDBS. 

### 3.4. The Optical Properties of OBC/DMDBS Samples

**Figure 7:**
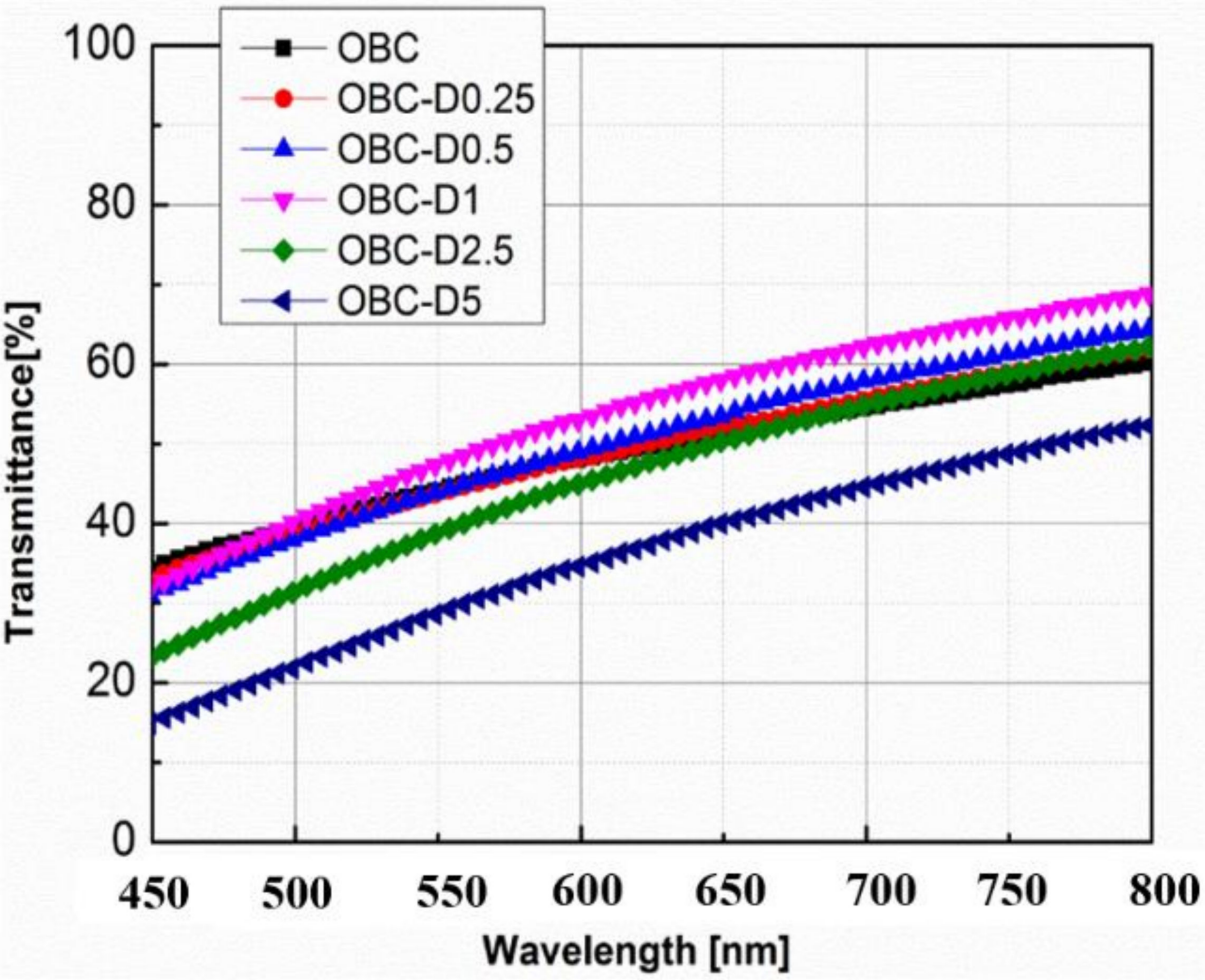
The optical properties of OBC/DMDBS mixtures containing different contents of DMDBS.

By adding a small amount of DMDBS, the transparency of the composites could be significantly affected. In general, decreased crystal size is favorable for enhancing optical transparency [39]. The optical properties of OBC composites with different content of DMDBS are compared in Figure 7. For the OBC/DMDBS system, the optical transparency of pure OBC was as good as ~30%. When the content of DMDBS was less than 1 wt %, the transparency of OBC/DMDBS composite was equal to that of pure OBC. With the increase of DMDBS content, the transparency of the composite gradually increased. However, when the content of DMDBS was higher than 1 wt %, the transparency of the sample decreased with the increase of DMDBS content. OBC-D5 showed a seriously decreased optical transparency. POM images have proven that DMDBS has a limited miscibility with OBC. When DMDBS content was larger than 1 wt %, DMDBS started to form aggregates and their size can even reach ~10μm. This was consistent with the optical properties of OBC/DMDBS samples. It can be concluded that a low addition of DMDBS (less than 1 wt %) acted as effective nucleation sites and greatly decreased the crystal size of OBC, which can strongly increase transparency, while microscale DMDBS aggregates induced by high additions of DMDBS caused deterioration of optical transparency.

### 3.5. The Mechanical Properties of OBC/DMDBS Samples

**Figure 8:**
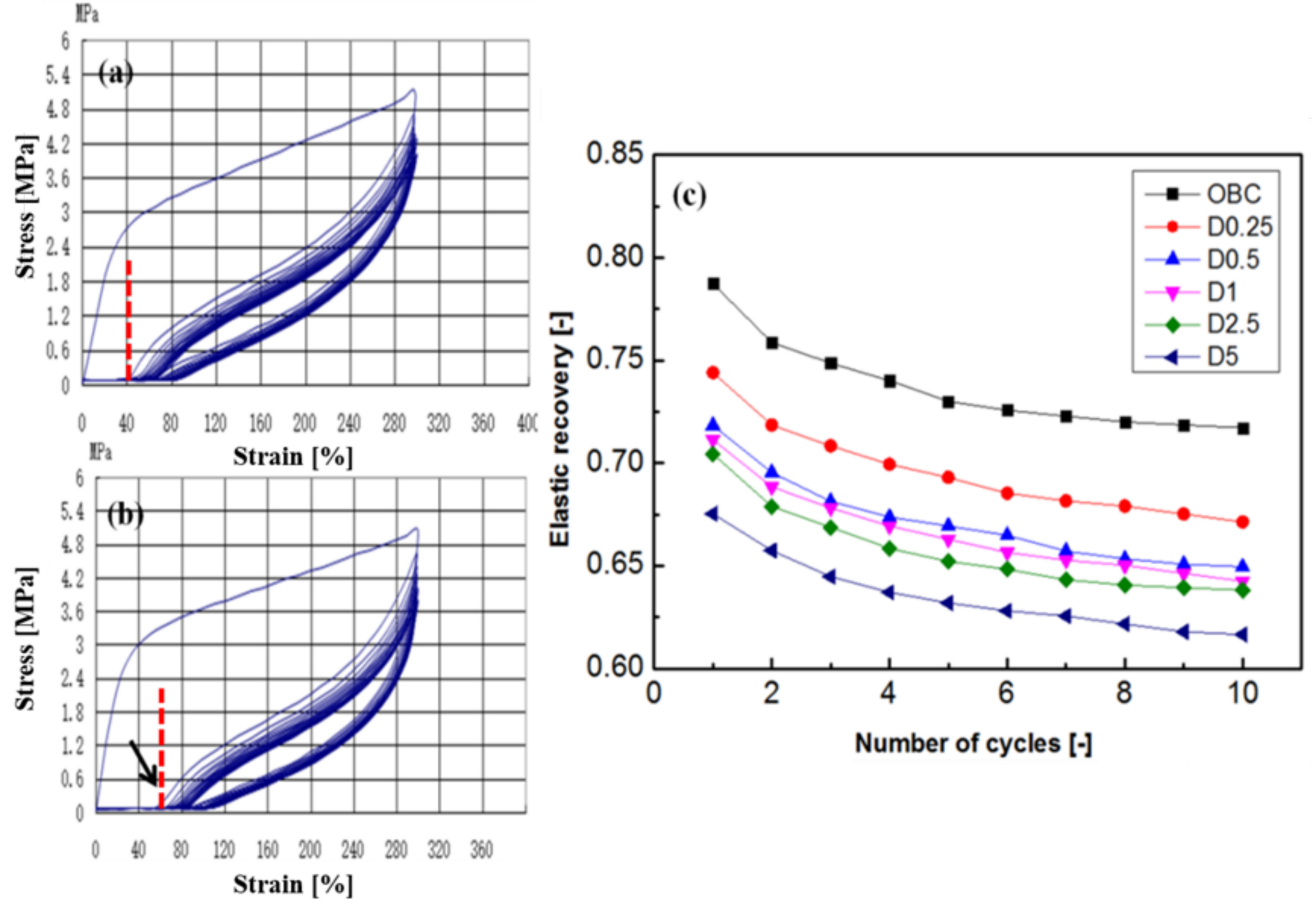
Representative cyclic stress-strain curves for OBC/DMDBS composites (**a**) neat OBC; and (**b**) OBC-D5; and (**c**) elastic recovery as a function of number of cycles for OBC/DMDBS with different contents of DMDBS.

Furthermore, the effect of DMDBS on the mechanical properties of OBC was also evaluated. The cyclic tensile properties of OBC/DMDBS composites are shown in Figure 8. During cyclic tensile deformation of pure OBC samples, the loading-unloading curve shows good elastomeric behavior of OBC/DMDBS samples. After adding DMDBS, the yielding stress increased from 2.7 MPa to 3.1 MPa. At the same time, the tensile stress of the sample decreased gradually with the increase of the number of tensile cycles, showing the phenomenon of stress softening, which is similar to the Mullins effect [39]. Most of the mechanical hysteresis and stress softening occurred in the first cycle of multiple cycles. It could be seen that OBC-D5 has a larger residual strain (63%) than that of neat OBC (42%), indicating that the introduction of DMDBS is unfavorable for elastic recovery. For detailed comparison, elastic recovery (ER) of these OBC samples was quantitatively evaluated by using the following equation [39]:

ER = (ε_max_ − ε(0,ε_max_))/ε_max_ (2)

where ε_max_ and ε (0,ε_max_) are the maximum strain and the strain in the cycle at zero stress after the maximum strain ε_max,_ respectively. Elastic recovery can reflect the ability to recover original shape after the removal of an external force. ER is generally less than 100%, meaning plastic deformation occurs during deformation. The elastic resilience of the OBC composites with different DMDBS content was compared. Figure 8c shows elastic recovery of OBC/DMDBS samples as a function of cycle numbers and DMDBS content. As shown in Figure 8c, ER values of the OBC/DMDBS composites decreased with the increase of DMDBS content or the increase of the cycle numbers. The elastic recovery of pure OBC remained above 70% after 10 cycles. However, when DMDBS reached 5 wt %, the elastic recovery of OBC-D5 was only 62% after 10 cycles. Hence, the introduction of DMDBS into OBC led to elastic loss, though yielding stress gradually increased with the increase of DMDBS content.

## 4. Conclusions

In summary, the influence of the addition of different contents of DMDBS on the crystallization behavior of OBC in OBC/DMDBS composites has been investigated. The crystallization behavior of OBC is strongly affected by the addition of DMDBS, which acts as nucleating sites and accelerates crystallization. With the increase of DMDBS content, the crystallization rate gradually increased, showing a strong composition dependence. OBC and OBC/DMDBS samples achieved typical spherulites. The introduction of DMDBS into OBC led to greatly decreased crystal size. Optical transparency can thus be improved. Due to limited miscibility, DMDBS formed aggregates within the OBC matrix when the content was high. In addition, OBC/DMDBS samples showed a slight increase of yielding stress, but suffered an elastic loss.

## Figures and Tables

**Table 1:** Melting temperature (T_m_), crystallization peak temperature (T_c,p_), enthalpy (ΔH_m_), and the relative crystallinity (X_c_) of OBC/DMDBS samples containing different content of DMDBS.

Sample	T_m_ [°C]	T_c,p_ [°C]	ΔH_m_ [J/g]	X_c_ [%]
**OBC**	121.4	106.3	45.362	15.6%
**D0.25**	121.8	107.7	45.914	15.8%
**D0.5**	122.0	108.9	47.119	16.1%
**D1**	122.1	110.9	45.954	15.8%
**D2.5**	122.6	113.9	44.441	15.3%
**D5**	122.8	114.0	42.862	14.7%
